# Miscellaneous Paragraphs

**Published:** 1893-11

**Authors:** 


### MISCELLANEOUS PARAGRAPHS.

### ANIMAL AND INVALID DIET.

Foods that will keep a well person healthy may kill the sick. On a diet of beef tea, which will build up an invalid, healthy men rapidly lose their strength. Rare, juicy beef, which is the most nutritive of all meats, and which nourishes the healthy, is the least nourishing of all foods for the sick person, whose feeble stomach can assimilate no part of it. The nutritive power of milk is very much underestimated. There is more nourishment in a pint of milk than there is in a quarter of a pound of beef. But this is not the whole question of invalid dieting. Chemistry has far less to do with the subject than the patient's stomach, which must have not what is most nourishing, but what it can assimilate with the least exertion. The food that a sick person likes and hungers for is invariably what nature requires. The perfect animal may be fed, the invalid must be fostered with simple but delicately serve morsels. The cheek of a broiled lamb chop, a checker of toast, a spoonful of wine jelly and an eggshell of hot milk—those are the dainties that provoke appetite.

---

### COLD HANDS AND FEET.

Many persons in poor health are troubled, even during the summer and early fall months, by cold hands and feet. These limbs should be educated, if possible, to keep themselves warm ; for certainly a cure cannot be effected by toasting them over a stove or a register, or by taking a hot bottle or bag to bed ; all these methods make them colder afterward. Primarily, the cause of this trouble is want of exercise, so all the exercise possible must be taken. But some one says : The more exercise I take, the colder I get. This is due to a reflex contraction of the blood vessels. The large nerves in the abdomen are connected with the blood vessels running down into the legs, by numbers of sympathetic nerves and branches, and when there is an irritation of the abdominal nerves, there is a contraction of the blood vassels, thus confusing the blood. When a person is nauseated, he is always pale. Why ? Because the irritation of the nerves of the stomach causes a contraction of the blood vessels. Now when a person is nauseated, he is not pale in the face only, but he is pale over the entire surface of the body ; there is not a contraction in the blood vessels only, but also in the brain ; there is not free blood enough to keep the heart going, so as to keep the person in his ordinary condition. There are thousands of chronic dyspeptics suffering from cold feet. This is caused by an irritation of the lumbar ganglia of the sympathetic nerves, situated close by the nerve trunks running into the legs ; it is a contraction or spasm of the blood vessels in the legs, induced by chronic irritation, that keeps the feet cold ; otherwise they would be kept warm by reason of the dependence of the limbs, as was intended by nature. Now it is not by exercise alone that this trouble can be cured ; the only way in which it can be permanently cured, is by relieving the abdominal irritation, and by relieving the dilated stomach ; that can be done by correcting the diet.

---

### HYSTERIA IN CHILDREN.

The cause of hysteria in children involves in most cases a history of a nervous disposition in the parents, this being frequently added to by debilitating diseases and bad feeling, anæmia and unfavorable moral surroundings. It may take the form of periodical outbursts of peculiar mental disturbance or may produce local symptoms—most commonly vague pains are complained of in various parts of the body, particularly in the joints, and both sides of the body are affected in the great majority of cases. Tremors are in some instances the first thing complained of, especially where traumatic influences have been at work. Spasms may occur in the muscles of speech and respiration, the speech becoming stammering or confused, or the patient be perfectly dumb for a longer or a shorter period ; the hysterical attacks are generally characterized by screaming, crying and laughing, accompanied by convulsive movements of the extremities, and sometimes slight loss of consciousness. The treatment which has proved most effectual is isolation, dashing cold water over the child, the faradic current and judicious verbal correction.

### A GLASS OF WATER AT BEDTIME.

The human body is constantly undergoing tissue change. Water has the power of increasing these tissue changes, which multiply the waste products, but at the same time they are renewed by its agency, giving rise to increased appetite, which in turn provides fresh nutriment. Persons but little accustomed to drink water are liable to have the waste products formed faster than they are removed. Any obstruction to the free working of natural laws at once produces disease. People accustomed to rise in the morning weak and languid will find the cause in the secretion of wastes, which many times may be remedied by drinking a full tumbler of water before retiring. This materially assists in the process during the night, and leaves the tissues fresh and strong, ready for the active work of the day. Hot water is one of the best remedial agents. A hot bath on going to bed, even in the hot nights of summer, is a better reliever of insomnia than many drugs.

### SHOULD THE SKINS OF FRUITS BE EATEN?

If the skins of fruits are to be eaten, they should be first purged of germs. The skins carry germs; the bloom of the peach and the grape is made up of germs,—luxuriant growths of microbes,—and when these skins are eaten, the microbes enter the stomach, and there they find one of the finest fields for growth in the world. The skin protects the fruit from the action of these germs. But if the skin is bruised or broken or injured in the slightest degree, the microbes get inside, and then the mischief begins. It is exactly the same as with the flesh of a human being; a little sliver will get under the skin, under the finger nail, and soon we will have an abrasion, a sore, and pus, and we will wonder that such a little thing could cause such a sore; the reason is that germs have found their way through that abrasion; pus-making germs are always to be found just under the edge of the finger nail, and when the sliver is thrust in there, it is equivalent to an inoculation of pus, for when the germs are carried in there, suppuration begins very quickly; whereas other portions of the body which are thoroughly cleansed would not be so liable to the action of germs. A rusty nail thrust into the foot will produce lockjaw in many cases ; and this is because, on the soiled nail there are often present germs of tetanus (germs which produce lockjaw), and the nail carries them througb the skin into the flesh. Now skins of fruit, being infested with germs, when eaten, carry those germs down into the stomach along with the fruit, and these germs then cause the fruit to decompose. Many persons are not able to eat raw fruit for this reason.

---

### DANGERS OF EATING FRESH BREAD.

New bread, in its smallest parts, is so soft clammy, flexible and glutinous, that by mastication it is with great difficulty separated and reduced to smaller parts, and is less under the influence of the saliva and gastric juices. It consequently forms itself into hard balls by careless and hasty mastication and deglutition, becomes coated over with saliva and slime, and in this state enters the stomach. The gastric juice being unable to penetrate such hard masses, and being scarcely able even to act upon the surface of them, they frequently remain in the stomach unchanged, and, like foreign bodies, irritate and incommode it, inducing every species of suffering,—oppression of the stomach, pain in the chest, disturbed circulation of the blood, congestion and pain in the head, irritation of the brain, and inflammation, apoplectic attacks, cramp and delirium.

